# Bovine Milk Oligosaccharides and Human Milk Oligosaccharides Modulate the Gut Microbiota Composition and Volatile Fatty Acid Concentrations in a Preclinical Neonatal Model

**DOI:** 10.3390/microorganisms9050884

**Published:** 2021-04-21

**Authors:** Mei Wang, Marcia H. Monaco, Jonas Hauser, Jian Yan, Ryan N. Dilger, Sharon M. Donovan

**Affiliations:** 1 Department of Food Science & Human Nutrition, University of Illinois, Urbana, IL 61801, USA; mewang@illinois.edu (M.W.); monaco@illinois.edu (M.H.M.); 2 Société des Produits Nestlé SA, 1000 Lausanne, Switzerland; Jonas.Hauser@rdls.nestle.com; 3 Nestlé Product Technology Center Nutrition, CH-1800 Vevey, Switzerland; jy435@cornell.edu; 4 Department of Animal Sciences and the Piglet Nutrition and Cognition Laboratory, University of Illinois, Urbana, IL 61801, USA; rdilger2@illinois.edu

**Keywords:** gut microbiota, milk oligosaccharide, volatile fatty acid, 2′fucosyllactose, lacto-N-neotetraose

## Abstract

Milk oligosaccharides (OS) shape microbiome structure and function, but their relative abundances differ between species. Herein, the impact of the human milk oligosaccharides (HMO) (2′-fucosyllactose [2′FL] and lacto-N-neotetraose [LNnT]) and OS isolated from bovine milk (BMOS) on microbiota composition and volatile fatty acid (VFA) concentrations in ascending colon (AC) contents and feces was assessed. Intact male piglets received diets either containing 6.5 g/L BMOS (n = 12), 1.0 g/L 2′FL + 0.5 g/L LNnT (HMO; n = 12), both (HMO + BMOS; n = 10), or neither (CON; n = 10) from postnatal day (PND) 2 to 34. Microbiota were assessed by 16S rRNA gene sequencing and real-time PCR, and VFA were measured by gas chromatography. The microbiota was affected by OS in an intestine region-specific manner. BMOS reduced (*p* < 0.05) microbial richness in the AC, microbiota composition in the AC and feces, and acetate concentrations in AC, regardless of HMO presence. HMO alone did not affect overall microbial composition, but increased (*p* < 0.05) the relative proportion of specific taxa, including *Blautia*, compared to other groups. *Bacteroides* abundance was increased (*p* < 0.05) in the AC by BMOS and synergistically by BMOS + HMO in the feces. Distinct effects of HMO and BMOS suggest complementary and sometimes synergistic benefits of supplementing a complex mixture of OS to formula.

## 1. Introduction

Breastfeeding is the recommended form of feeding human infants and is associated with both short- and long-term health benefits [1,2]. Exclusive breastfeeding is recommended by the American Academy of Pediatrics for the first 6 months of life [3]; however, only 25% of American infants meet this recommendation [4]. As such, 75% of babies in the U.S. are either exclusively formula-fed or are receiving both human milk (HM) and infant formula by 6 months of age [4]. Thus, adapting infant formula composition to more closely resemble to composition of HM has been an active area of on-going research [5].

While commercial infant formula have undergone significant modifications over the past few decades with the additional of bioactive ingredients, such as prebiotics, lactoferrin, and milk fat globule membrane [6], dissimilarities in human milk and infant formula persist, with the content and composition of oligosaccharides (OS) constituting one of the largest compositional differences. Human milk oligosaccharides (HMO) constitute the third most abundant solid component in human milk, after lactose and fat. They are present in concentrations ranging from 20 to 25 g/L in colostrum (the first milk produced by female mammals immediately after giving birth) and 5 to 15 g/L in mature milk and have great structural diversity, with up to 200 structures having been identified [7,8]. In contrast, bovine milk, the most common starting material for infant formula, contains only trace amounts of OS (~1 to 2 g/L in colostrum and 100 mg/L in mature milk), with less complex structures (30–50 structures identified) [9,10]. Three major categories of OS have been identified in human milk: neutral fucosylated (e.g., 2′-fucosyllactose, 2′-FL), neutral nonfucosylated (e.g., lacto-N-neotetraose, LNnT), and acidic (e.g., 3′-sialyllactose, 3′-SL, and 6′-sialyllactose, 6′-SL), and some structures that are both fucosylated and sialylated [11]. Compared with bovine milk, human milk contains a larger proportion of fucosylated structures (50–80% vs. ∼1%) and smaller proportion of sialylated structures (10–20% vs. ∼70%) [7].

Several studies have reported beneficial effects of feeding HMO and bovine milk derived oligosaccharides (BMOS), including modulation of growth [12], alkaline phosphatase activity [13], gene transcription [14], and barrier function [15,16,17] of the intestinal epithelial cell in vitro. HMO also influence the development of the immune system [18,19] and the brain [20,21] and shape the development of the infant gut microbiota. [22,23]. Different HMO function as prebiotics by stimulating the growth of beneficial bacteria, such as *Bifidobacterium*, while suppressing potential pathogens [8,24,25,26,27]. Lastly, due to their structural similarity with mucosal glycans, HMO and BMOS act as soluble decoy receptor, inhibiting the adhesion of pathogenic microorganisms or bacterial toxins to the host cell receptors [25]. For these reasons, they have been recommended to be added to infant formula [22,27].

To date, two HMO, 2′-FL and LNnT, are commercially available and have been added to infant formula [28,29]; up to now, there is no commercial sources of synthesized HMO that fully represent the breadth of milk OS present in human milk [30]. Due to the lack of diversity in OS structures, infant formula based on bovine milk is unlikely to recapitulate all the functions of human milk when it comes to the OS fraction. Several studies have demonstrated that BMOS purified from whey permeate (a by-product obtained when cheese whey is passed through an ultrafiltration membrane to concentrate whey protein) have some structural features in common with HMO [31], suggesting OS isolated from bovine milk may provide some beneficial effects associated with HMO.

Due to the high degree of similarity in anatomy, physiology, immunology, and brain development patterns between pigs and humans, piglets are considered an ideal model for neonatal nutrition research [32,33,34]. Piglets have been extensively used as a preclinical model to study the effects of diet and prebiotics on microbiome development [20,21,33,34,35,36,37]. Therefore, the aim of the study was to evaluate the effect of HMO (2′FL + LNnT), BMOS, and a combination of BMOS and HMO on gut microbiota composition and volatile fatty acid (VFA) concentrations in a piglet model. We hypothesized that supplementation of HMO and/or BMOS to formula would modulate the gut microbiota composition and their metabolic products, and that HMO and BMOS would act in synergy when added to formula.

## 2. Materials and Methods

### 2.1. Animal Care and Dietary Treatments

The study design and diets composition were previously described by Fleming et al. [34]. Briefly, naturally farrowed, intact male piglets (n = 44) were obtained from a commercial swine farm and transferred to the Piglet Nutrition and Cognition Laboratory, University of Illinois, at postnatal day (PND) 2. The piglets were randomized to four diets: control (CON; Purina Pro Nurse Specialty Milk, Purina Animal Nutrition, St. Louis, MO, USA; n = 10), BMOS (CON + 6.5 g/L BMOS; n =12), HMO (CON + 1.0 g/L 2′FL + 0.5 g/L LNnT; n = 12) or HMO + BMOS (CON + 6.5 g/L BMOS + 1.0 g/L 2′FL + 0.5 g/L LNnT; n = 10). All diets were supplemented with lactose to equalize the added carbohydrate to 8 g/L in the reconstituted milk replacer that was provided to piglets. The nutritional composition of the base formula and oligosaccharide content have been previously reported [34]. The OS concentrations were chosen to remain consistent with previously conducted clinical trials on 2′-FL and LNnT [28,29] and BMOS [38,39,40,41].

BMOS was generated from whey permeate of bovine milk containing galactooligosaccharides (GOS) and other OS from bovine milk, such as 3′- and 6′-sialyllactose [38]. The OS composition of BMOS was previous reported (described as GMOS in [42]. 2′FL and LNnT were supplied by Glycom A/S (Kongens Lyngby, Denmark). Milk replacer powder was reconstituted fresh each day at 200 g of dry powder per 800 g of water. Piglets were weighed every morning and provided 285 and 325 mL of reconstituted milk replacer treatment/kg BW from PND 2–6 and PND 7–33, respectively. Milk was automatically delivered to piglets on a set schedule, with 10 equally-spaced meals per day. The study was completed in six replicates (6–8 pigs per replicate). Piglets were housed in custom pig rearing units in the same room with a 12 h light/dark cycle. All animal care and experimental procedures were in accordance with National Research Council Guide for the Care and Use of Laboratory Animals and approved by the University of Illinois at Urbana-Champaign Institutional Animal Care and Use Committee.

### 2.2. Sample Collection

Ascending colon (AC) contents and feces were collected on PND 34. AC contents were collected as most dietary OS are fermented in the colon. Fecal samples were also studied as the microbiota composition of colon contents differed from that of feces [42] and studies conducted in healthy term human infants rely on fecal samples. Fecal samples were obtained by inserting a wet cotton swab into the rectum of the animal to stimulate muscular movement. For AC content sampling, piglets were sedated with 7 mg/kg BW Telazol (Fort Dodge Animal Health, Fort Dodge, IA, USA) and then euthanized by intra-cardiac injection of 72 mg/kg BW sodium pentobarbital (Fatal Plus, Vortech Pharmaceuticals, Dearborn, MI, USA). The large intestine was isolated and separated into cecum and colon at the cecocolic junction. The colon was further divided equally into the ascending, transverse and descending colon [35]. For study of microbiota, AC contents and feces were collected into sterile cryogenic tubes, snap-frozen in liquid nitrogen, and stored at −80 °C. For VFA analysis, AC contents and feces were mixed with 2N HCl (1000:1 *w/v* ratio) in Eppendorf tubes and stored at −20 °C until analyses were performed.

### 2.3. Dry Matter, pH, and VFA Concentrations

Dry matter (DM), pH, and VFA (acetate, propionate, butyrate, isobutyrate, valerate, and isovalerate) concentrations were measured on AC contents and feces. Dry matter was assessed on the samples according to the AOAC method [35]. pH was measured using an Orion 2-star pH meter and electrode (Thermo Fisher Scientific, Waltham, MA, USA). VFA concentrations were determined by gas chromatography as previously described [35]. Data were expressed as μmol/g of dry matter.

### 2.4. DNA Extraction, PCR Amplification and Sequencing of 16S rRNA Genes

DNA was isolated from AC contents and feces by combination of the QIAamp Fast DNA Stool Mini Kit (Qiagen, Valencia, CA, USA) with bead beating on the FastPrep-24 System (MP Biomedicals, Carlsbad, CA, USA), as previously described [36]. The concentration of DNA was measured with a NanoDrop 1000 spectrophotometer (NanoDrop Technologies, Wilmington, DE, USA). PCR amplification and sequencing of the V3–V4 region of 16S rRNA genes were performed at the DNA Services Lab, University of Illinois as described by Monaco et al. [37].

### 2.5. Sequence Processing

Sequences were demultiplexed at the sequencing facility with the bcl2fastq v2.17.1.14 Conversion Software (Illumina, San Diego, CA, USA), allowed 0 mismatches in the barcode sequences. De-multiplexed forward and reverse reads were processed using the QIIME pipeline (version 1.9.1) [43]. Briefly, the paired-end reads were merged, quality filtered, and split into libraries as described by Monaco et al. [37]. Operational taxonomic units (OTU) assignment, representative sequence picking, chimera removing, sequence alignment, and phylogenetic tree construction were performed as previously described [17]. Taxonomic assignment for each representative sequence was done using Ribosomal Database Project naïve Bayesian rRNA Classifier at 80% confidence level on the Greengenes reference database (gg_13_8_otus/taxonomy/97_otu_taxonomy.txt, accessed on 24 February 2017) [44]. An OTU table was generated and further filtered to remove non-aligned and chimeric OTUs and singletons. Alpha diversity (observed OTUs, Chao1 and Shannon and Simpson reciprocal indices) and unweighted UniFrac distances were calculated from the filtered OTU table after rarefying to an equal number of reads (10,500) to standardize the sampling effort.

### 2.6. Real-Time PCR

Real-time PCR was used as a complementary approach to sequencing in order to study the effect of dietary treatment on opportunistic pathogenic species that are associated with infection in infants, including Enterobacteriaceae, *Bacteroides fragilis*, *Clostridium perfringens*, *Clostridium difficile*, and *Escherichia coli* [45,46,47], as sequencing V3–V4 region of 16S cannot not accurately classified all sequences into species level. We quantified *Bifidobacterium* spp. and *Lactobacillus* spp., as members of these genera utilize milk OS [48] and provide beneficial effect on human health. *Prevotella* spp. contains enzymes involved in mucin oligosaccharide degradation [49] and an increase in *Prevotella* abundance was associated with high fiber intake [50]. Real-time PCR was performed in QuantStudio 7 Flex System (Thermo Fisher Scientific, Waltham, MA, USA) using TaqMan (for *B. fragilis* and *E. coli*) or SYBR Green (for other bacterial groups/species) assays as previously described [37] using primers/probes listed in Table S1. The standard curves were generated using 10-fold dilution of purified plasmid DNA (10–10^8^ 16S rRNA gene copies/reaction). Data analysis was processed with QuantStudio software V1.3 supplied by Thermo Fisher Scientific (Waltham, MA, USA) and presented as 16S rRNA gene copy numbers/g of AC contents or feces.

### 2.7. Statistics

All data were analyzed as 2 × 2 factorial design. Differences in overall bacterial community structure among dietary treatments were evaluated with distanced-based redundancy analysis (db-RDA). db-RDA were performed on unweighted UniFrac distances using capscale command of Vegan package of R [51]. The statistical mode included HMO, BMOS and interaction of HMO and BMOS. db-RDA is a constrained ordination method that performs redundancy analysis (RDA) on orthogonal principal coordinates (PCOs), which is obtained from principal coordinates analysis (PCoA) using a distance measure (unweighted UniFrac distance in this study). db-RDA has been widely used in microbial ecology studies [23,52]. Univariate statistical analysis (real-time PCR, alpha-diversity, relative abundances of bacterial taxa, pH, DM, and VFA) was performed using the PROC MIXED procedure of SAS version 9.4. The model included the main (HMO, and BMOS) and interactive effects of HMO and BMOS as fixed effects, and replicate as a random effect (SAS Institute, Cary, NC, USA). Tukey post hoc tests were applied when the interaction is significant. When data were not normally distributed, arcsine-square root (relative abundance of bacterial taxa) or log10 (real-time PCR and VFA) transformations were applied to normalize the residual distribution. Data were reported as means ± SEM unless specially indicated. Statistical significance was set at *p* < 0.05.

## 3. Results

### 3.1. DM, pH, and VFA Concentrations

The percent dry matter was lower in piglets fed either BMOS containing diets (BMOS and HMO + BMOS groups) than in piglets consumed diets without BMOS (CON and HMO groups) in both AC contents and feces (Table 1; *p* = 0.0025 and *p* = 0.0136, respectively). HMO supplementation had no effect on AC or fecal DM and pH (Table 1; *p*-values for HMO > 0.05). The addition of BMOS to the diets increased the acetate concentrations in AC regardless of the presence of HMO (*p* = 0.0473). Fecal acetate concentration was highest in HMO + BMOS and CON, intermediate in BMOS and lowest in HMO piglets, while butyrate concentrations were highest in HMO + BMOS, intermediate in CON and BMOS and lowest in HMO (Table 1; *p* < 0.05). Neither HMO nor BMOS affected propionate, isobutyrate, valerate or isovalerate concentrations at either sampling location.

### 3.2. Sequencing of 16S rRNA Amplicons

Illumina sequencing of 16S rRNA amplicons yielded 9.1 million paired-end reads (107,075 ± 3913 per sample) across the 85 samples. After assembling of paired-end reads, quality filtering and removing of singletons, 3,970,157 sequences (46,707 ± 1921 per sample) were utilized for further analysis.

**Figure 1:**
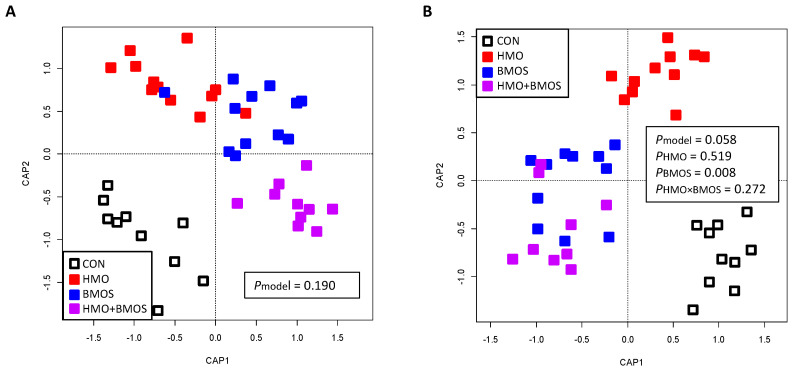
Distance-based redundancy analysis based on unweighted UniFrac distances generated from ascending colon contents (**A**) and feces (**B**) of piglets fed different oligosaccharide-containing diets. The statistical model included HMO, BMOS and the interaction of HMO and BMOS (HMO × BMOS). There was no significant differences between the diet groups in the ascending colon (*P*_model_ = 0.190). There was a trend (*P*_model_ = 0.058) for an effect of diet in the feces, with BMOS-containing diets (BMOS and HMO + BMOS) clustering separately from diets without BMOS (CON and HMO). CAP: constrained analysis of principal coordinates.

**Figure 2:**
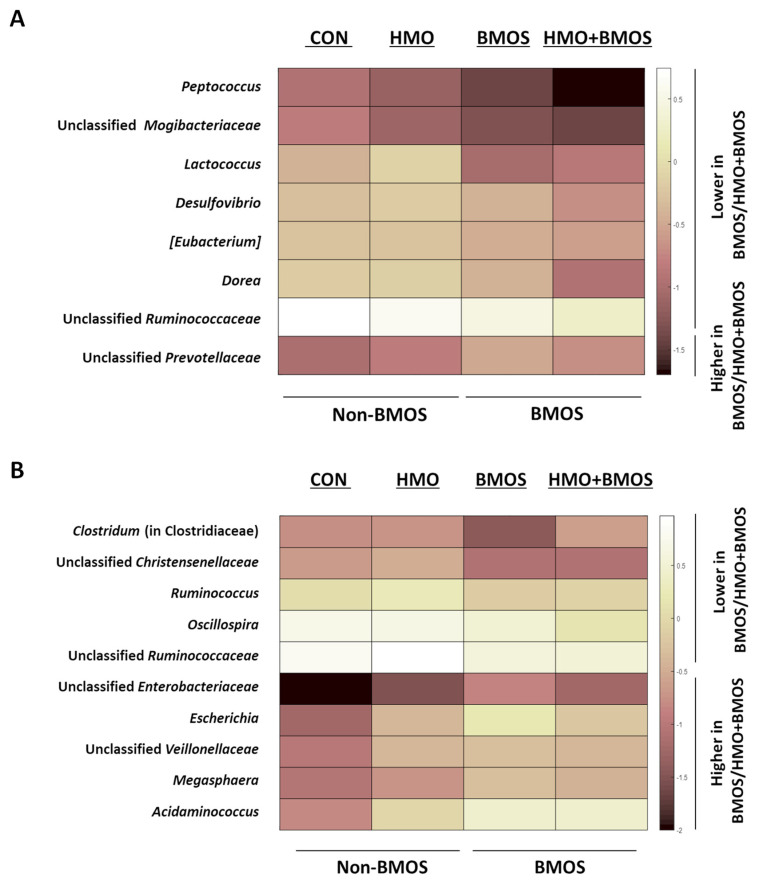
Effect of BMOS-containing diets on the relative abundances of bacterial genera in the ascending colon (**A**) and feces (**B**) of piglets fed different oligosaccharide-containing diets. Relative abundances of 7 bacterial genera were lower and 1 was higher in ascending colon of piglets fed diets with BMOS (BMOS and HMO + BMOS groups) than without BMOS (CON and HMO groups; *p* < 0.05) (**A**). Relative abundances of 10 bacteria genera differed between piglets consuming diets with BMOS (BMOS and HMO + BMOS groups) and without BMOS (CON and HMO groups) in feces (*p* < 0.05) (**B**). Data for the heat maps were log10 transformed.

Distance-based redundancy analysis based on unweighted Unifrac distances indicated that the addition of BMOS (BMOS and BMOS + HMO groups) did not affect the overall microbiota composition in the AC contents when both BMOS and HMO were included in the statistical model (Figure 1A; *P*_model_ = 0.190); however, a significant effect of BMOS was observed (*p* = 0.012) when BMOS was considered as the only main effect (Figure S1). Differential abundance analysis of bacterial phyla and genera showed proportions of some bacterial taxa were impacted by BMOS. Bacteroidetes were higher (Table 2; *p* = 0.0432) and Tenericutes (Table 3; *p* = 0.0032) were lower in the AC of piglets consuming diets with BMOS (BMOS and HMO + BMOS groups) than without BMOS (CON and HMO groups). At the genus level, BMOS supplementation decreased relative abundances of *Dorea*, *Eubacterium*, *Peptococcus*, *Lactococcus*, *Desulfovibrio*, and unclassified Mogibacteriaceae, whereas it increased unclassified Preovotellaceae in the AC (Figure 2A, Table S1; *p* < 0.05).

**Table 1:** pH, dry matter and volatile fatty acid concentrations of AC contents and feces from piglets fed different oligosaccharide-containing diets.

	CON (n = 10)	HMO (n = 10–11)	BMOS (n = 8–11)	HMO + BMOS (n = 6–8)	*p*-Values		
					HMO	BMOS	HMO × BMOS
**Ascending colon**							
pH	6.67 ± 0.16	6.86 ± 0.08	6.80 ± 0.10	6.51 ± 0.18	0.7063	0.4082	0.0558
DM (%)	20.1 ± 1.82	19.5 ± 1.69	16.6 ± 1.17	16.3 ± 1.31	0.3431	0.0025	0.4405
Acetate (μmol/g of DM)	439.6 ± 99.8	423.3 ± 98.2	511.4 ± 90.2	512.4 ± 113.5	0.3873	0.0473	0.4746
Propionate (μmol/g of DM)	138.1 ± 31.4	125.2 ± 30.5	128.4 ± 21.2	128.0 ± 24.2	0.5545	0.5030	0.6591
Butyrate (μmol/g of DM)	98.4 ± 28.5	59.2 ± 9.79	71.3 ± 12.2	69.7 ± 14.9	0.5667	0.9412	0.8236
Isobutyrate (μmol/g of DM)	9.34 ± 1.52	10.1 ± 2.22	9.30 ± 1.23	8.91 ± 1.29	0.4518	0.8059	0.2631
Valerate (μmol/g of DM)	11.5 ± 1.66	12.3 ± 2.45	10.8 ± 1.04	10.3 ± 0.82	0.6290	0.1346	0.1890
Isovalerate (μmol/g of DM)	17.8 ± 3.95	16.4 ± 3.25	16.2 ± 2.13	17.2 ± 3.16	0.5977	0.5838	0.4524
**Feces**							
pH	6.89 ± 0.12	6.99 ± 0.09	7.13 ± 0.08	7.07 ± 0.07	0.7818	0.0587	0.3066
DM (%)	32.3 ± 2.56	36.5 ± 1.97	28.7 ± 2.13	27.9 ± 3.30	0.4698	0.0136	0.3040
Acetate (μmol/g of DM)	146.1 ± 21.0 ^a^	95.4 ± 9.59 ^b^	132.6 ± 19.5 ^ab^	180.8 ± 43.5 ^a^	0.5653	0.1044	0.0394
Propionate (μmol/g of DM)	42.4 ± 5.60	29.9 ± 4.10	40.2 ± 7.17	49.5 ± 10.3	0.7676	0.1521	0.0831
Butyrate (μmol/g of DM)	20.0 ± 2.62 ^ab^	13.0 ± 2.46 ^b^	14.9 ± 3.07 ^ab^	36.7 ± 19.0 ^a^	0.9446	0.3624	0.0291
Isobutyrate (μmol/g of DM)	5.81 ± 1.01	4.85 ± 0.77	5.61 ± 0.65	6.24 ± 1.30	0.7980	0.4364	0.3902
Valerate (μmol/g of DM)	8.49 ± 1.85	7.20 ± 1.41	7.70 ± 0.85	9.31 ± 2.30	0.8394	0.3726	0.4515
Isovalerate (μmol/g of DM)	5.57 ± 0.93	4.67 ± 0.98	5.32 ± 0.93	6.66 ± 1.38	0.8564	0.1345	0.2303

Values are means ± SEMs. *p*-values were obtained using PROC MIXED procedure of SAS with HMO, BMOS, and interaction of HMO and BMOS (HMO × BMOS) as fixed effects and replicate as a random effect. Tukey post hoc test was applied when the interaction is significant. ^a,b^ When the interaction is significant, labeled means in a row without common superscript differ, *p* < 0.05. BMOS, diet with bovine milk oligosaccharides alone; CON, control diet; DM, dry matter; HMO, diet with human milk oligosaccharides alone; HMO + BMOS, diet with both HMO and BMOS. Supplementation with BMOS modulated the overall fecal bacterial composition (*p* = 0.008; Figure 1B). Compared with piglets fed diets without BMOS (CON and HMO groups), piglets that consumed BMOS (BMOS and HMO + BMOS groups) had lower proportions of fecal Tenericutes (Table 3; *p* = 0.0292). At the genus level, relative abundances of *Escherichia*, *Megasphaera*, *Acidaminococcus*, and unclassified Veilonellaceae and Enterobacteriaceae were higher, while *Oscillospira*, *Ruminococcus*, and *Clostridium* in the family of Clostridiaceae, and unclassified Christensenellaceae and Ruminococcaceae were lower (Figure 2B, Table S2; *p* < 0. 05) in feces of piglets fed BMOS-containing diets (BMOS and HMO + BMOS groups) compared to piglets fed diets without BMOS (CON and HMO groups).

**Table 2:** Relative abundances of bacterial phyla in ascending colon of piglets fed different oligosaccharide-containing diets.

Phylum	CON (n = 10)	HMO (n = 12)	BMOS (n = 12)	HMO + BMOS (n = 10)	*p*-Values		
					HMO	BMOS	HMO × BMOS
Actinobacteria	0.81 ± 0.23	1.84 ± 0.89	0.84 ± 0.37	0.42 ± 0.15	0.7297	0.1451	0.2349
Bacteroidetes	42.8 ± 8.73	37.8 ± 6.88	47.7 ± 5.73	55.5 ± 5.73	0.7864	0.0432	0.3107
Cyanobacteria	0.01 ± 0.01	0.31 ± 0.30	0 ± 0	0 ± 0	0.3223	0.2774	0.3181
Deferribacteres	0.06 ± 0.04 ^a^	0.01 ± 0.01 ^b^	0.01 ± 0 ^b^	0.04 ± 0.03 ^ab^	0.2511	0.394	0.0158
Elusimicrobia	1.62 ± 0.66	1.10 ± 0.58	2.04 ± 1.21	3.14 ± 2.24	0.9516	0.6119	0.5118
Firmicutes	46.4 ± 8.70	53.1 ± 6.89	50.0 ± 5.51	36.4 ± 5.90	0.8138	0.1056	0.2419
Fusobacteria	2.58 ± 2.38	0.03 ± 0.02	0.04 ± 0.02	0.08 ± 0.07	0.1553	0.1809	0.1444
Lentisphaerae	0.01 ± 0.01	0 ± 0	0.01 ± 0	0.01 ± 0	0.1712	0.5105	0.2735
Proteobacteria	1.93 ± 0.53	2.08 ± 0.41	1.67 ± 0.41	2.37 ± 1.32	0.6862	0.6198	0.9967
Synergistetes	0.05 ± 0.03	0.09 ± 0.07	0.03 ± 0.02	0.04 ± 0.02	0.5248	0.416	0.9753
Tenericutes	0.38 ± 0.22	0.07 ± 0.02	0.11 ± 0.09	0.02 ± 0.02	0.0170	0.0032	0.3194
Verrucomicrobia	0.07 ± 0.05	1.04 ± 1.03	0.26 ± 0.17	0.02 ± 0.01	0.9169	0.8424	0.3211
Unclassified	1.36 ± 0.17	1.30 ± 0.13	1.07 ± 0.06	1.09 ± 0.11	0.8083	0.0266	0.7509

**Figure 3:**
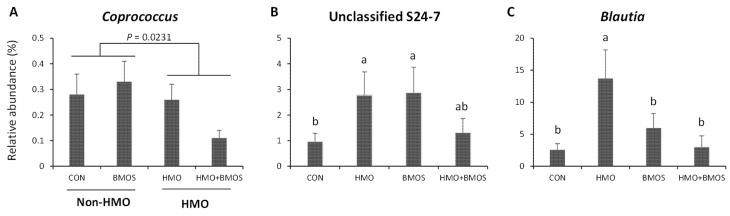
Relative abundances of ascending colon bacterial genera that differed by diet. *Coprococcus* was higher in piglets fed diets without HMO (CON and BMOS groups) compared to diets with HMO (HMO and HMO + BMO groups) (**A**). Compared to the CON diet, Unclassified S24-7 was greater in piglets fed diets containing HMO or BMOS alone, with the HMO + BMOS diet being intermediate (**B**). *Blautia* was greater in piglets fed the HMO alone than all other diet groups (**C**). Values are means ± SEM. ^a,b^ Labeled means without a common letter differ, *P* (BMOS and HMO + BMOS groups) compared to piglets fed diets without BMOS (CON and HMO groups; *p* < 0.05). The proportion of *Blautia* was higher in HMO than all other groups and unclassified S24-7 were highest in piglets fed either HMO or BMOS, intermediate in HMO + BMOS, and lowest in CON piglets (Figure 3B,C; *p* < 0.05).

Values are means ± SEMs. *S*-values were obtained using PROC MIXED procedure of SAS with HMO, BMOS, and the interaction of HMO and BMOS (HMO × BMOS) as fixed effects and replicate as a random effect. Tukey post hoc tests was applied when interaction was significant. ^a,b^ When the interaction is significant, labeled means within a row without a common superscript differ, *p* < 0.05. BMOS, diet with bovine milk oligosaccharides alone; CON, control diet; HMO, diet with human milk oligosaccharides alone; HMO + BMOS, diet with both HMO and BMOS. Supplementation with HMO had no effect on overall bacterial community structure of the AC contents or feces (*p* = 0.886 and *p* = 0.519, respectively; Figure 1A,B). However, the relative abundances of bacterial phyla and genera indicated that proportions of several bacterial taxa were significantly affected by HMO supplementation (Tables S2 and S3). Phylum Tenericutes was lower in both AC and feces of piglets fed diets with HMO (HMO and HMO + BMOS groups) than without HMO (CON and BMOS groups) (*p* = 0.0170 and *p* = 0.0313, respectively; Table 2 and Table 3). Genus *Coprococcus* was lower in AC of piglets fed diets with HMO (HMO and HMO + BMOS groups) than without HMO (CON and BMOS groups; *p* = 0.023; Figure 3A).

**Table 3:** Relative abundances of bacterial phyla in feces of piglets fed different oligosaccharide-containing diets.

Phylum	CON (n = 10)	HMO (n = 11)	BMOS (n = 11)	HMO + BMOS (n = 9)	*p*-Values		
					HMO	BMOS	HMO × BMOS
Actinobacteria	0.92 ± 0.32	2.56 ± 0.93	1.10 ± 0.50	1.06 ± 0.45	0.1788	0.2220	0.1324
Bacteroidetes	49.5 ± 5.73 ^ab^	39.6 ± 5.47 ^b^	44.2 ± 4.0 ^ab^	54.11 ± 3.60 ^a^	0.9400	0.2825	0.0317
Cyanobacteria	0.01 ± 0.01	0.04 ± 0.04	0 ± 0	0 ± 0	0.2377	0.1500	0.4995
Deferribacteres	0.02 ± 0.01	0 ± 0	0.02 ± 0.01	0 ± 0	0.1123	0.9305	0.8006
Elusimicrobia	4.02 ± 2.31	6.58 ± 3.19	4.13 ± 1.69	5.93 ± 2.59	0.5138	0.8268	0.8141
Firmicutes	37.1 ± 4.43	44.1 ± 5.69	41.3 ± 4.88	33.6 ± 3.40	0.8313	0.3986	0.0711
Fusobacteria	2.38 ± 2.00	0.60 ± 0.38	1.03 ± 0.75	0.11 ± 0.05	0.1906	0.2594	0.8083
Lentisphaerae	0.09 ± 0.06	0.08 ± 0.03	0.04 ± 0.02	0.02 ± 0.01	0.8342	0.0778	0.5231
Proteobacteria	2.39 ± 0.94	1.73 ± 0.35	3.54 ± 0.96	1.77 ± 0.44	0.1124	0.3798	0.3321
Synergistetes	0.45 ± 0.19	1.31 ± 0.87	0.34 ± 0.12	0.76 ± 0.47	0.2246	0.3505	0.5413
Tenericutes	0.26 ± 0.16	0.03 ± 0.01	0.05 ± 0.03	0.02 ± 0.01	0.0313	0.0292	0.0789
Verrucomicrobia	1.06 ± 0.50	1.51 ± 1.35	2.02 ± 1.16	0.49 ± 0.36	0.3031	0.9871	0.5431
Unclassified	1.50 ± 0.18	1.47 ± 0.13	1.83 ± 0.17	1.46 ± 0.16	0.1817	0.3248	0.2708

Values are means ± SEMs. *p*-values were obtained using PROC MIXED procedure of SAS with HMO, BMOS and the interaction of HMO and BMOS (HMO × BMOS) as fixed effects and replicate as a random effect. Tukey post hoc test was applied when the interaction is significant. ^a,b^ When the interaction is significant, labeled means in a row without a common superscript differ, *p* < 0.05. BMOS, diet with bovine milk oligosaccharides alone; CON, control diet; HMO, diet with human milk oligosaccharides alone; HMO + BMOS, diet with both HMO and BMOS.

The effects of BMOS and HMO on alpha diversity are summarized in Table 4. Observed OTUs (*p* = 0.0148), Chao1 (*p* = 0.0321) and Faith PD (*p* = 0.0066) were lower in AC of piglets fed diets with BMOS (BMOS and HMO + BMOS groups) than diets without BMOS (CON and HMO groups). A similar effect on Chao1 was also observed in AC of piglets fed HMO-containing diets (HMO and HMO + BMOS) (*p* = 0.0275). In feces, observed OTUs. Chao1 and Faith PD did not differ among the diet groups (*p* > 0.05); however, Simpson reciprocal index was highest in BMOS, intermediate in HMO and HMO + BMOS, and lowest in CON group (*p* < 0.05). Neither HMO nor BMOS supplementation affected Shannon index (*p* > 0.05).

### 3.3. RT PCR

**Figure 4:**
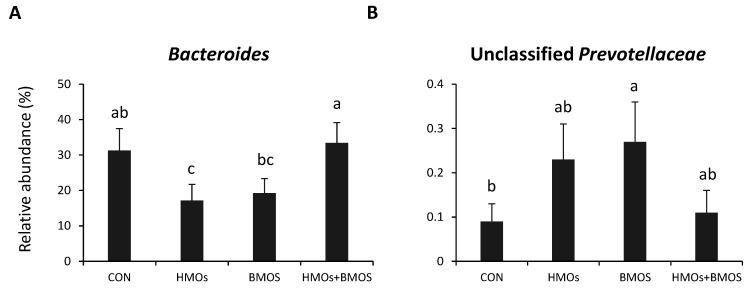
Relative abundances of fecal bacterial genera that differed by diet. *Bacteroides* was reduced in piglets fed HMO alone compared to CON and HMO + BMOS; BMOS differed from HMO + BMOS (**A**). Unclassified Prevotellaceae was greater in BMOS than CON, with HMO-containing diets (HMO and HMO + BMO groups) being intermediate (**B**). Values are means ± SEM. ^a,b,c^ Labeled means without a common letter differ, *p* < 0.05. In feces, the relative abundances of Bacteroidetes were highest in HMO + BMOS, intermediate in CON and BMOS and lowest in HMO piglets (Table 3; *p* < 0.05). At the genus level, the proportion of fecal *Bacteroides* was highest in HMO + BMOS and lowest in HMO, while unclassified Prevotellaceae were highest in BMOS, intermediate in HMO and HMO + BMOS and lowest in CON piglets (Figure 4A,B; *p* < 0.05).

Real-time PCR results are presented in Table 5. Densities of *Prevotella* spp. in AC and Enterobacteriaceae and *E. coli* in feces were higher in piglets fed diets containing BMOS (BMOS, HMO + BMOS) than diets without BMOS (CON and HMO; *p* < 0.05). HMO had no effect on abundances of any bacterial groups/species measured by RT PCR (*p* > 0.05) (Figure 4).

**Table 4:** Alpha diversity obtained from microbiota of ascending colon and feces of piglets fed different oligosaccharide-containing diets.

	CON (n = 10)	HMO (n = 11–12)	BMOS (n = 11–12)	HMO + BMOS (n = 9–10)	*p*-Values		
					HMO	BMOS	HMO × BMOS
**Ascending colon**							
Observed OTUs	2123.2 ± 124.1	2014.2 ± 100.6	1922.3 ± 105.6	1777.0 ± 84.0	0.1358	0.0148	0.8187
Shannon	7.24 ± 0.37	7.10 ± 0.25	6.96 ± 0.31	6.54 ± 0.25	0.2829	0.1172	0.5843
Simpson reciprocal	27.12 ± 7.90	18.47 ± 2.48	20.74 ± 2.97	14.98 ± 2.69	0.2674	0.4494	0.6410
Chao1	5527.6 ± 310.8	5003.3 ± 227.5	5019.3 ± 314.6	4521.6 ± 179.8	0.0275	0.0321	0.9991
Faith PD	153.98 ± 8.00	150.70 ± 7.18	141.99 ± 5.47	132.47 ± 6.45	0.2075	0.0066	0.5543
**Feces**							
Observed OTUs	2230.3 ± 77.0	2187.3 ± 119.6	2354.7 ± 73.3	2048.7 ± 149.8	0.0793	0.9205	0.2156
Shannon	7.66 ± 0.17	7.72 ± 0.20	7.96 ± 0.19	7.36 ± 0.32	0.2037	0.8925	0.1217
Simpson reciprocal	24.68 ± 2.49 ^b^	31.74 ± 4.19 ^ab^	37.47 ± 4.70 ^a^	27.35 ± 4.94 ^ab^	0.7041	0.2999	0.0377
Chao1	6051.6 ± 248.6	5841.7 ± 389.1	6315.6 ± 245.9	5527.4 ± 406.6	0.0948	0.9096	0.3951
Faith PD	165.19 ± 4.69	166.45 ± 7.48	174.55 ± 5.58	152.40 ± 9.73	0.0903	0.6571	0.0832

Values are means ± SEMs. *p*-values were obtained using PROC MIXED procedure of SAS with HMO, BMOS and the interaction between HMO and BMOS (HMO × BMOS) as fixed effects and replicate as a random effect. Tukey post hoc test was applied when the interaction is significant. ^a,b^ When the interaction is significant, labeled means in a row without common superscript differ, *p* < 0.05. BMOS, diet with bovine milk oligosaccharides alone; CON, control diet; DM, dry matter; HMO, diet with human milk oligosaccharides alone; HMO + BMOS, diet with both HMO and BMOS; OTU, operational taxonomic unit; PD, phylogenetic diversity.

**Table 5:** Bacterial densities in the ascending colon contents and feces of piglets fed different oligosaccharide-containing diets.

	CON (n = 10)	HMO (n = 11–12)	BMOS (n = 11–12)	HMO + BMOS (n = 9–10)	*p*-Values		
					HMO	BMOS	HMO × BMOS
	Log_10_ 16S rRNA gene copies/g of AC contents or feces						
**Ascending colon**							
Total bacteria	11.0 ± 0.07	11.1 ± 0.07	11.0 ± 0.05	11.2 ± 0.09	0.1612	0.2765	0.5490
Enterobacteriaceae	8.82 ± 0.28	8.91 ± 0.32	9.38 ± 0.18	9.12 ± 0.30	0.7559	0.1306	0.4818
*Bifidobacterium* spp.	6.73 ± 0.27	7.07 ± 0.17	7.23 ± 0.18	7.02 ± 0.15	0.5418	0.1384	0.1283
*Lactobacillus* spp.	9.69 ± 0.15	9.64 ± 0.12	9.54 ± 0.18	9.81 ± 0.17	0.4833	0.9444	0.1796
*Prevotella* spp.	10.6 ± 0.25	10.6 ± 0.20	10.9 ± 0.20	11.1 ± 0.16	0.5011	0.0210	0.4291
*Bacteroides fragilis*	6.57 ± 0.52	6.53 ± 0.38	6.40 ± 0.32	6.31 ± 0.31	0.7790	0.5282	0.9274
*Clostridium difficile*	4.89 ± 0.07	4.88 ± 0.08	4.92 ± 0.13	5.14 ± 0.20	0.4131	0.2323	0.3541
*Clostridium perfringens*	6.81 ± 0.31	6.47 ± 0.29	6.32 ± 0.30	6.10 ± 0.40	0.3708	0.1840	0.8425
*Escherichia coli*	8.14 ± 0.32	8.29 ± 0.35	8.80 ± 0.20	8.45 ± 0.32	0.7617	0.1365	0.3564
**Feces**							
Total bacteria	10.4 ± 0.17	10.3 ± 0.24	10.6 ± 0.10	10.6 ± 0.11	0.8045	0.2313	0.4963
Enterobacteriaceae	8.31 ± 0.16	8.51 ± 0.40	9.42 ± 0.24	8.97 ± 0.25	0.6544	0.0039	0.2356
*Bifidobacterium* spp.	6.02 ± 0.20	6.47 ± 0.24	6.46 ± 0.26	6.62 ± 0.24	0.1849	0.2021	0.5273
*Lactobacillus* spp.	8.98 ± 0.17	9.51 ± 0.31	9.48 ± 0.15	9.13 ± 0.30	0.5456	0.1502	0.1827
*Prevotella* spp.	10.4 ± 0.22	10.3 ± 0.16	10.6 ± 0.16	10.6 ± 0.15	0.8392	0.2374	0.6492
*Bacteroides fragilis*	6.45 ± 0.45	6.38 ± 0.35	5.99 ± 0.31	5.92 ± 0.36	0.8413	0.2076	0.9962
*Clostridium difficile*	4.91 ± 0.08	4.89 ± 0.09	5.09 ± 0.17	4.88 ± 0.06	0.2542	0.3929	0.3663
*Clostridium perfringens*	5.82 ± 0.39	5.93 ± 0.44	5.74 ± 0.36	5.18 ± 0.20	0.5237	0.2476	0.3579
*Escherichia coli*	7.51 ± 0.16	7.69 ± 0.46	8.75 ± 0.26	8.26 ± 0.27	0.6217	0.0035	0.2863

Values are means ± SEMs. *p*-values were obtained using PROC MIXED procedure of SAS with HMO, BMOS and the interaction between HMO and BMOS (HMO × BMOS) as fixed effects and replicate as a random effect. BMOS, diet with bovine milk oligosaccharides alone; CON, control diet; DM, dry matter; HMO, diet with human milk oligosaccharides alone; HMO + BMOS, diet with both HMO and BMOS. *C. perfringens*, *C. difficile*, and *B. fragilis* were not detected in all the samples. When the abundances were lower than detection limit (1.25 × 10^5^ copies of 16S rRNA genes/g for *C. difficile* and *C. perfringens*, and 3.13 × 10^5^ copies/g for *B. fragilis*), ½ value of the detection limit was used for statistical analysis.

## 4. Discussion

Colonization of gut microbiota plays an essential role in host metabolism, development of neonatal gastrointestinal, immune, and neural systems, and also affects short- and long-term health outcomes [53,54]. The gut microbiota is established during the first 2–3 y of life and influenced by host genetics and environmental factors, such as prenatal exposures, gestational age, delivery method, feeding mode; and pre-, pro-, and antibiotic use, of which feeding mode plays a major determinant role [55,56,57,58,59]. Despite efforts to narrow the compositional differences between human milk and infant formula, differences in the gut microbial composition between breastfed and formula fed infants persist [6,23,56,59]. The presence of high concentrations of structurally diverse OS in human milk has motivated efforts to supplement infant formula initially with prebiotics, and more recently with 2′FL and/or LNnT in an effort to drive the microbiota composition of formula-fed infants closer to breastfed infants [19,28,59]. In our study, two HMOs (2′FL and LNnT), BMOS, and a combination of these HMOs and BMOS were added to formula. Using the neonatal pig model, we found that BMOS- and HMO-supplementation affected the gut bacterial composition. Furthermore, synergistic effects were observed on some bacterial populations when a combination of HMO and BMOS were added to formula.

Although bovine milk contains lower concentration of OS than human milk, the two share at least 10 common structures [60,61]. Both contain large amounts of the acidic OS, such as 3′SL and 6′SL [31]. Due to their identical structures, it is expected that OS derived from bovine milk would have similar functional properties of HMO [60,61]. Recently, BMOS were commercially purified from whey permeate [31,61] and intervention studies have shown BMOS-supplemented infant formulas (alone or in combination with probiotics) were well tolerated and supported normal growth of healthy term infants [38,40]. Meli et al. [38] investigated the effects of BMOS on the stool bacterial counts using culture plating and fluorescence in situ hybridization (FISH) and reported that clostridia counts was lower in infants consumed BMOS supplemented formula compared to the unsupplemented group. Both plate counting and FISH allow for the estimation of the abundance of specific groups/species of bacteria; however, they do not provide an untargeted view on the microbiota.

In this study, high-throughput sequencing was applied to assess the effect of BMOS on the global profile of gut microbial communities. We demonstrated BMOS altered overall bacterial community structures and modulated the relative abundances of bacterial taxa in both AC contents and feces. Effects of BMOS on microbiota differed at the 2 intestinal sampling sites. For instance, BMOS increased the abundances of *Prevotella* spp. in AC contents, but not in feces. *Prevotella* spp. is one of the three major genera that differentiated individual gut microbiota into enterotypes [62]. Previous studies have shown high levels of *Prevotella* were associated with long-term high fiber intake in humans [50]. In our study, BMOS lowered alpha diversity (observed OTUs, Chao1 and Faith PD) in AC, while it had no effect on microbial diversity in feces. Several reasons could explain the different effects of BMOS in AC contents and feces. Previous studies have shown the microbiota composition and functions of in the colon differed from that of stool in both humans and mice [42] and different gut bacteria consumed milk OS differently [24]. Furthermore, BMOS contains mainly short-chain OS (GOS, 3′SL and 6′SL) and carbohydrate fermentation primarily occurred in AC of piglets, leaving less subtracts for fecal microbiota to utilize [63].

Similarly, BMOS-containing formula elicited significant effects on VFA concentrations. The VFA are bacterial fermentation end-products of dietary fibers and amino acids and their profiles represent the collective metabolic activity of the gut microbiota. OS are minimally digested by hydrolytic/enzymatic digestion in the upper gastrointestinal tract and most dietary OS reach the colon where they are subsequently fermented by the gut microbiota [24]. In our study, concentrations of acetate were significantly greater in AC contents of piglets fed diets with BMOS (BMOS, BMOS + HMO groups) than without BMOS (CON and HMO groups), indicating supplementation with BMOS changes not only the composition, but also the metabolic function, of gut microbiota of piglets. While the roles of VFA in the infant gastrointestinal tract development are still being investigated, in vitro data showed that acetate contributed to acidification of the intestinal milieu and inhibited the growth of many common pathogens [64]. Additionally, up to 70% of the acetate is taken up by the liver cells, where they act as substrates for the synthesis of cholesterol, long-chain fatty acids, and the amino acids glutamine and glutamate [65].

The HMO, 2′FL and LNnT, are highly abundant in human milk. 2′FL is the predominant oligosaccharide in the milk of secretor mothers, representing nearly 30% of total HMO with a mean level of 2–3 g/L [66]. The concentrations of LNnT in mature human milk have been reported in the range of 0.1 to 0.6 g/L [28]. A double-blind randomized controlled multicenter trial demonstrated that formula supplemented with 2′FL (1 g/L) and LNnT (0.5 g/L) was safe, well-tolerated, and supported normal growth of healthy term infants. Additionally, infants receiving formula with those two HMO had fewer parent-reported adverse events, particularly bronchitis [28]. More recently, Berger and colleagues [29] examined the effects of 2′FL and LNnT on the infant fecal microbiota composition at 3 mo of age, reporting that infants fed formula with 2 HMO developed a distinctive microbial colonization patterns that were closer to breastfed infants than infants fed unsupplemented formula. Supplementation with 2′FL and LNnT reduced alpha diversity (Faith’s diversity index) and increased the proportion of the genus *Bifidobacterium* to a level closer to breastfed infants [29].

In contrast with the results of human infant study, neither Faith PD nor overall bacterial structure were impacted by HMO supplementation in the piglets in this study. Furthermore, relative abundances of *Bifidobacterium* were low in piglets and there were no difference among the dietary groups. These contradictory observations likely arise from the fundamental differences in the gut microbiota composition of infants and piglets. The predominant phyla of piglets are Bacteroidetes and Firmicutes [37,67,68], which is more similar to that of the human adult [69]. Actinobacteria, predominantly *Bifidobacterium*, is generally dominant in infant stool microbiota regardless of how they are fed. While *Bifidobacterium* is detectable in the gastrointestinal tract of piglets, the relative abundance is considerably lower (<0.1% of 16S rRNA gene sequences) than in infants. Furthermore, the *Bifidobacterium* species detected in piglets differed from that of human infants. *B. longum* subsp. *infantis*, *B. longum* subsp. *longum*, *B. breve*, *B. catenulatum*, and *B. adolescentis* represent the predominant species of human infants, while *Bifidobacterium* found in piglets are *B. longum* subsp. *suis*, *B. globosum* and *B. pseudolongum* [70,71]. A recent review on the prevalence of HMO-utilization genes in bifidobacterial genomes, reported that HMO assimilation abilities differ among *Bifidobacterium* species and strains [72]. Additionally, HMO-related genes are almost exclusively found in the genomes of infant gut-associated *Bifidobacterium* species, and hardly detected in the genomes of *Bifidobacterium* species isolated from human adults and animals [72]. We have shown that piglets can be colonized with *B. longum* subsp. *infantis* [36], thus future studies in piglets colonized with human infant microbiota are warranted to better understand the physiological potential of HMO in neonates [73].

The relative abundances of *Blautia* were higher in the AC contents of piglets supplemented with HMO alone compared to other groups. *Blautia* is a genus in the bacterial family Lachnospiraceae that phylogenetically belongs to *Clostridium* Cluster XIVa within phylum Firmicutes [74,75]. *Blautia* is widely distributed among fecal samples of human and animals [8,76]. Members of *Blautia* are efficient degraders of dietary fibers and producers of short chain fatty acids [76]. Several studies have suggested that intestinal *Blautia* may play a role in human health outcomes. Compared to healthy children, the abundance of *Blautia* was significantly lower in the fecal microbiota of children with autism spectrum disorders, but higher in children with type 1 diabetes [77,78]. Despite their apparent importance, little is known about their presence and the possible roles played by *Blautia* spp. in humans during the neonatal period, warranting further studies.

Human milk contains both neutral and acidic OS. 2′FL and LNnT are neutral OS, while BMOS are rich in acidic OS such as 3′SL and 6′SL. The biological activities of HMO are dependent upon their chemical structures, we hypothesized that the combination of HMO and BMOS would more closely mimic the structural complexity and biological functions of OS present in the human milk. We observed synergistic effects on gut microbiota composition when HMO + BMOS were supplemented to formula. The relative abundance of fecal Bacteroides was 33% higher in piglets receiving BMOS + HMO than piglets consuming BMOS (19%) or HMO (17%) alone. *Bacteroides* is a predominant genus found in the gut of human adults, while its abundance varies greatly in infant feces with some infants being dominated by *Bacteroides* rather than *Bifidobacterium* [79]. In vitro studies have demonstrated that some members of *Bacteroides* are effective consumers of milk OS [24,80] and an inverse correlation was observed in the proportion of fecal *Bifidobacterium* and *Bacteroides* in breastfed infants [23,80]. In this study, *Bacteroides* was the predominant bacterial genus in both AC contents and feces of piglets; whereas the relative abundances of *Bifidobacterium* are very low (<0.1% of sequences). This is perhaps not a surprising result as genomic analysis of *Bacteroides* spp. revealed a specialized machinery encoded by the polysaccharide utilization loci (PULs) dedicated to the import and processing of HMO, plant polysaccharides and mucins [80]. As previous discussed, *Bifidobacterium* species isolated from pig, including *B. globosum* and *B. pseudolongum*, lack HMO-utilizing genes [71]. Therefore, when *Bifidobacterium* spp. cannot utilize milk OS, supplementation of HMO + BMOS provides a selective advantage for *Bacteroides* growth in the gut of piglets. Given human milk contains both OS and bacteria and that microbiota of vaginally-delivered breastfed infants is typically predominated by *Bifidobacterium* [81,82], our results suggest that if the intent of OS supplementation is to modulate infant microbial populations, the OS should be paired with *Bifidobacterium* that avidly consumes OS, especially for infants whose early microbiota are dominated by other bacteria, such as *Bacteroides* and Enterobacteriaceae, and not with HMO-utilizing *Bifidobacterium* [82].

## 5. Conclusions

Supplementation of BMOS and BMOS + HMO modulated microbiota composition and VFA profiles in the neonatal model investigated herein. The addition of HMO did not affect overall bacterial communities; however, HMO alone increased the proportion of specific taxa, such as *Blautia*. Synergistic effects, such as an increase in *Bacteroides*, were observed when combination of HMO and BMOS were added to formula. Taken together with the observed OS-specific effects on recognition memory, both absolute and relative volumes of cortical and subcortical brain regions, and hippocampal mRNA expression in these same piglets [34] demonstrate that HMO and BMOS exert distinct actions and formula-fed infants could benefit from formula containing a variety of neutral, fucosylated and sialylated milk OS.

## Data Availability

Data will be available upon request.

## Supplementary Materials

The following supporting materials are available online at https://www.mdpi.com/article/10.3390/microorganisms9050884/s1, Table S1. Real-time PCR primers and probe used to determine density of microbiota in ascending colon contents and feces. Table S2. Relative abundances of bacterial genera detected in AC of piglets fed different diets. Table S3. Relative abundances of bacterial genera detected in feces of piglets fed different diets. Figure S1. Distance-based redundancy analysis based on unweighted UniFrac distances generated from AC contents of piglets fed diets with or without BMOS.
