# Supplementary material for: Bovine Milk Oligosaccharides and Human Milk Oligosaccharides Modulate the Gut Microbiota Composition and Volatile Fatty Acid Concentrations in a Preclinical Neonatal Model
Source: Microorganisms. 2021 Apr 21;9(5):884. doi: 10.3390/microorganisms9050884 (PMC8143120; doi:10.3390/microorganisms9050884)
Supplement: Supplementary file 1 [file microorganisms-09-00884-s001.zip › microorganisms-1169313 suppl final.pdf]

**Table S1.** Real-time PCR primers and probe used to determine density of microbiota in ascending colon contents and feces.

| Target group | Primer/Probe | Sequence (5'-3') | Annealing temperature, °C | Reference |
|---|---|---|---|---|
| All bacteria | Uni331F<br>Uin797R | TCCTACGGGAGGCAGCAGT<br>GGACTACCAGGGTATCTATCCTGTT | 60 | Nadkarni et al., 2002 |
| Enterobacteriaceae | Eco1457F<br>Eco1652R | CATTGACGTTACCCGCAGAAGAAGC<br>CTCTACGAGACTCAAGCTTGC | 63 | Bartosch et al., 2004 |
| *Bifidobacterium* spp. | Bif164F<br>Bif662R | GGGTGGTAATGCCGGATG<br>CCACCGTTACACCGGGAA | 60 | Kok et al., 1996 |
| *Lactobacillus* spp. | LacF<br>LacR | AGCAGTAGGGAATCTTCCA<br>CACCGCTACACATGGAG | 58 | Rinttila et al., 2004 |
| *Prevotella* spp. | R-prevF<br>R-prevR | GGTTCTGAGAGGAAGGTCCCC<br>TCCTGCACGCTACTTGGCTG | 60 | Stevenson et al. 2007 |
| *Bacteroides fragilis* | Bfragilis_F<br>Bfragilis_R<br>BF_Probe* | TCRGGAAGAAAGCTTGCT<br>CATCCTTTACCGGAATCCT<br>ACACGTATCCAACCTGCCCTTTACTCG | 56 | Tong et al., 2011 |
| *Clostridium difficile* | Cdiff_F<br>Cdiff_R | TTGAGCGATTTACTTCGGTAAAGA<br>CCATCCTGTACTGGCTCACCT | 58 | Rinttila et al. 2004 |
| *Clostridium perfringens* | s-Clper-F<br>ClPER-R | GGGGGTTTCAACACCTCC<br>GCAAGGGATGTCAAGTGT | 60 | Matsuda et al., 2009 |
| *Escherichia coli* | Ecoli_F<br>Ecoli_R<br>Ecoli_Probe* | CATGCCGCGTGTATGAAGAA<br>CGGGTAACGTCAATGAGCAAA<br>ATTAACTTTACTCCCTTCCTCCCCGCTGAA | 60 | Pender et al., 2005 |

*Probe has FAM label on the 5′ end, minor groove binder and nonfluorescent quencher on the 3′ end.

**Table S2.** Relative abundances of bacterial genera detected in AC contents of piglets fed different diets[1,2].

| Genus | Family | Phylum | CON (*n* = 10) | HMO (*n* = 12) | BMOS (*n* = 12) | HMO+BMOS (*n* = 10) | *P* value | | |
|---|---|---|---|---|---|---|---|---|---|
| | | | | | | | HMO | BMOS | HMO×BMOS |
| Collinsella | Coriobacteriaceae | Actinobacteria | 0.09 ± 0.03 | 0.15 ± 0.06 | 0.09 ± 0.04 | 0.09 ± 0.05 | 0.3802 | 0.2662 | 0.4190 |
| Uncla Coriobacteriaceae | Coriobacteriaceae | Actinobacteria | 0.59 ± 0.21 | 1.58 ± 0.83 | 0.66 ± 0.34 | 0.27 ± 0.11 | 0.6441 | 0.1637 | 0.2497 |
| Butyricimonas | [Odoribacteraceae] | Bacteroidetes | 0.54 ± 0.17 | 0.54 ± 0.17 | 0.52 ± 0.15 | 0.58 ± 0.26 | 0.9328 | 0.9680 | 0.8456 |
| [Prevotella] | [Paraprevotellaceae] | Bacteroidetes | 0.83 ± 0.60 | 0.99 ± 0.57 | 0.92 ± 0.53 | 0.70 ± 0.27 | 0.8469 | 0.9241 | 0.7695 |
| Uncla [Paraprevotellaceae] | [Paraprevotellaceae] | Bacteroidetes | 0.69 ± 0.27 | 2.35 ± 1.65 | 1.29 ± 0.73 | 1.67 ± 0.62 | 0.2794 | 0.8651 | 0.7997 |
| Bacteroides | Bacteroidaceae | Bacteroidetes | 31.27 ± 8.50 | 20.00 ± 5.71 | 29.89 ± 5.53 | 36.04 ± 6.78 | 0.7210 | 0.1539 | 0.1372 |
| Parabacteroides | Porphyromonadaceae | Bacteroidetes | 2.04 ± 0.90 | 1.04 ± 0.22 | 1.75 ± 0.59 | 4.23 ± 2.58 | 0.7414 | 0.3331 | 0.2509 |
| Prevotella | Prevotellaceae | Bacteroidetes | 2.65 ± 2.26 | 6.12 ± 3.01 | 6.38 ± 2.70 | 6.16 ± 3.13 | 0.4970 | 0.1993 | 0.4447 |
| Uncla Prevotellaceae | Prevotellaceae | Bacteroidetes | 0.10 ± 0.04 | 0.15 ± 0.05 | 0.32 ± 0.09 | 0.20 ± 0.06 | 0.9353 | **0.0271** | 0.2909 |
| Alistipes | Rikenellaceae | Bacteroidetes | 0.26 ± 0.09 | 0.17 ± 0.06 | 0.15 ± 0.05 | 0.19 ± 0.09 | 0.6073 | 0.3828 | 0.3152 |
| Uncla Rikenellaceae | Rikenellaceae | Bacteroidetes | 0.88 ± 0.40 | 0.63 ± 0.24 | 0.51 ± 0.17 | 0.89 ± 0.51 | 0.9133 | 0.5666 | 0.3946 |
| Uncla S24-7 | S24-7 | Bacteroidetes | 0.96 ± 0.33[b] | 2.77 ± 0.91[a] | 2.87 ± 0.99[a] | 1.31 ± 0.55[ab] | 0.5654 | 0.6451 | **0.0062** |
| Uncla Elusimicrobiaceae | Elusimicrobiaceae | Elusimicrobia | 1.62 ± 0.66 | 1.10 ± 0.58 | 2.04 ± 1.21 | 3.14 ± 2.24 | 0.9514 | 0.6121 | 0.5119 |
| Uncla [Mogibacteriaceae] | [Mogibacteriaceae] | Firmicutes | 0.15 ± 0.05 | 0.08 ± 0.02 | 0.05 ± 0.02 | 0.04 ± 0.02 | 0.1347 | **0.0026** | 0.4694 |
| Clostridium | Clostridiaceae | Firmicutes | 0.11 ± 0.06 | 0.10 ± 0.04 | 0.24 ± 0.14 | 0.36 ± 0.28 | 0.9446 | 0.4856 | 0.8948 |
| [Eubacterium] | Erysipelotrichaceae | Firmicutes | 0.53 ± 0.16 | 0.53 ± 0.18 | 0.36 ± 0.13 | 0.27 ± 0.10 | 0.5360 | **0.0424** | 0.6468 |
| Catenibacterium | Erysipelotrichaceae | Firmicutes | 0.10 ± 0.10 | 0.21 ± 0.14 | 0.03 ± 0.03 | 0 ± 0 | 0.8514 | 0.1472 | 0.3222 |
| RFN20 | Erysipelotrichaceae | Firmicutes | 0.03 ± 0.01 | 0.01 ± 0.01 | 0.04 ± 0.02 | 0.72 ± 0.62 | 0.2388 | 0.0836 | 0.0831 |
| Uncla Erysipelotrichaceae | Erysipelotrichaceae | Firmicutes | 0.12 ± 0.05 | 0.08 ± 0.02 | 0.05 ± 0.01 | 0.07 ± 0.05 | 0.6481 | 0.0855 | 0.8990 |
| [Ruminococcus] | Lachnospiraceae | Firmicutes | 2.84 ± 0.56 | 5.24 ± 2.01 | 6.07 ± 2.05 | 6.29 ± 1.60 | 0.3136 | 0.1440 | 0.6406 |
| Blautia | Lachnospiraceae | Firmicutes | 2.58 ± 0.94[b] | 13.72 ± 4.46[a] | 5.97 ± 2.27[b] | 2.97 ± 1.78[b] | 0.1134 | 0.1339 | **0.0032** |
| Coprococcus | Lachnospiraceae | Firmicutes | 0.28 ± 0.08 | 0.26 ± 0.06 | 0.33 ± 0.08 | 0.11 ± 0.03 | **0.0231** | 0.3376 | 0.0770 |
| Dorea | Lachnospiraceae | Firmicutes | 0.65 ± 0.24 | 0.68 ± 0.29 | 0.37 ± 0.12 | 0.11 ± 0.05 | 0.2593 | **0.0287** | 0.3598 |
| Uncla Lachnospiraceae | Lachnospiraceae | Firmicutes | 3.37 ± 0.86 | 4.45 ± 0.77 | 5.38 ± 0.96 | 3.71 ± 0.65 | 0.6750 | 0.4229 | 0.0771 |
| Lactobacillus | Lactobacillaceae | Firmicutes | 12.44 ± 5.53 | 9.14 ± 3.66 | 11.68 ± 3.41 | 11.82 ± 3.91 | 0.7363 | 0.8210 | 0.6009 |
| Uncla Lactobacillaceae | Lactobacillaceae | Firmicutes | 0.70 ± 0.39 | 0.29 ± 0.07 | 0.24 ± 0.07 | 0.20 ± 0.08 | 0.2847 | 0.0811 | 0.3870 |
| Leuconostoc | Leuconostocaceae | Firmicutes | 3.00 ± 2.86 | 1.08 ± 0.57 | 0.14 ± 0.06 | 0.29 ± 0.24 | 0.9042 | 0.1238 | 0.4828 |
| Uncla Leuconostocaceae | Leuconostocaceae | Firmicutes | 0.08 ± 0.06 | 0.09 ± 0.05 | 0.02 ± 0.01 | 0.03 ± 0.02 | 0.8693 | 0.0940 | 0.8018 |

**Continued**

**Table S2.** Relative abundances of bacterial genera detected in AC contents of piglets fed different diets (continued)[1,2].

| Genus | Family | Phylum | CON ($n$ = 10) | HMO ($n$ = 12) | BMOS ($n$ = 12) | HMO+BMOS ($n$ = 10) | *P* value | | |
|---|---|---|---|---|---|---|---|---|---|
| | | | | | | | HMO | BMOS | HMO×BMOS |
| Peptococcus | Peptococcaceae | Firmicutes | 0.11 ± 0.05 | 0.07 ± 0.03 | 0.04 ± 0.02 | 0.02 ± 0.01 | 0.3461 | **0.0083** | 0.8592 |
| Anaerotruncus | Ruminococcaceae | Firmicutes | 0.08 ± 0.02 | 0.05 ± 0.02 | 0.06 ± 0.02 | 0.10 ± 0.06 | 0.6923 | 0.9343 | 0.2551 |
| Butyricicoccus | Ruminococcaceae | Firmicutes | 0.14 ± 0.04 | 0.23 ± 0.07 | 0.11 ± 0.04 | 0.17 ± 0.09 | 0.2355 | 0.3481 | 0.7527 |
| Faecalibacterium | Ruminococcaceae | Firmicutes | 2.48 ± 0.85 | 2.25 ± 0.69 | 2.35 ± 0.99 | 0.83 ± 0.32 | 0.1357 | 0.0876 | 0.2791 |
| Oscillospira | Ruminococcaceae | Firmicutes | 1.99 ± 0.16 | 1.68 ± 0.55 | 1.78 ± 0.48 | 1.34 ± 0.48 | 0.1104 | 0.2613 | 0.8937 |
| Ruminococcus | Ruminococcaceae | Firmicutes | 0.46 ± 0.13 | 0.44 ± 0.12 | 0.40 ± 0.14 | 0.41 ± 0.24 | 0.6618 | 0.4462 | 0.8858 |
| Uncla Ruminococcaceae | Ruminococcaceae | Firmicutes | 5.57 ± 1.38 | 4.15 ± 0.68 | 2.79 ± 0.85 | 1.79 ± 0.45 | 0.1160 | **0.0002** | 0.9415 |
| Lactococcus | Streptococcaceae | Firmicutes | 0.39 ± 0.21 | 0.78 ± 0.44 | 0.09 ± 0.04 | 0.13 ± 0.11 | 0.8018 | **0.0471** | 0.6939 |
| Streptococcus | Streptococcaceae | Firmicutes | 0.11 ± 0.04 | 0.07 ± 0.02 | 0.06 ± 0.02 | 0.10 ± 0.05 | 0.8463 | 0.3415 | 0.6381 |
| Acidaminococcus | Veillonellaceae | Firmicutes | 0.19 ± 0.13 | 0.60 ± 0.26 | 0.78 ± 0.23 | 0.53 ± 0.19 | 0.4480 | 0.0755 | 0.1088 |
| Anaerovibrio | Veillonellaceae | Firmicutes | 0.20 ± 0.20 | 0.17 ± 0.15 | 0.03 ± 0.03 | 0.31 ± 0.30 | 0.5044 | 0.7514 | 0.5848 |
| Dialister | Veillonellaceae | Firmicutes | 0.01 ± 0 | 0.06 ± 0.02 | 0.08 ± 0.03 | 0.09 ± 0.05 | 0.4004 | 0.0690 | 0.2669 |
| Megasphaera | Veillonellaceae | Firmicutes | 0.19 ± 0.07 | 0.31 ± 0.06 | 0.33 ± 0.08 | 0.24 ± 0.06 | 0.8532 | 0.5746 | 0.0900 |
| Mitsuokella | Veillonellaceae | Firmicutes | 0.15 ± 0.15 | 0.25 ± 0.17 | 0.03 ± 0.02 | 0.10 ± 0.10 | 0.3614 | 0.3142 | 0.6337 |
| Phascolarctobacterium | Veillonellaceae | Firmicutes | 0.06 ± 0.01 | 0.06 ± 0.01 | 0.08 ± 0.01 | 0.05 ± 0.01 | 0.1741 | 0.4714 | 0.0622 |
| Uncla Veillonellaceae | Veillonellaceae | Firmicutes | 0.26 ± 0.11 | 0.33 ± 0.12 | 0.32 ± 0.05 | 0.31 ± 0.09 | 0.9257 | 0.4733 | 0.3618 |
| Uncla Fusobacteriaceae | Fusobacteriaceae | Fusobacteria | 2.53 ± 2.33 | 0.03 ± 0.01 | 0.04 ± 0.02 | 0.08 ± 0.07 | 0.1584 | 0.1781 | 0.1447 |
| Sutterella | Alcaligenaceae | Proteobacteria | 0.20 ± 0.11 | 0.14 ± 0.10 | 0.15 ± 0.06 | 0.14 ± 0.06 | 0.1609 | 0.5366 | 0.5842 |
| Campylobacter | Campylobacteraceae | Proteobacteria | 0.52 ± 0.52 | 0.07 ± 0.04 | 0 ± 0 | 0.01 ± 0.01 | 0.6450 | 0.1395 | 0.5291 |
| Bilophila | Desulfovibrionaceae | Proteobacteria | 0.07 ± 0.04 | 0.06 ± 0.05 | 0.03 ± 0.01 | 0.04 ± 0.02 | 0.8756 | 0.5808 | 0.9352 |
| Desulfovibrio | Desulfovibrionaceae | Proteobacteria | 0.50 ± 0.13 | 0.64 ± 0.16 | 0.39 ± 0.09 | 0.20 ± 0.03 | 0.6388 | **0.0044** | 0.1362 |
| Uncla Desulfovibrionaceae | Desulfovibrionaceae | Proteobacteria | 0.10 ± 0.04 | 0.06 ± 0.01 | 0.06 ± 0.02 | 0.05 ± 0.01 | 0.2536 | 0.4004 | 0.6558 |
| Escherichia | Enterobacteriaceae | Proteobacteria | 0.25 ± 0.12 | 0.84 ± 0.41 | 0.84 ± 0.40 | 1.56 ± 1.29 | 0.5277 | 0.2788 | 0.5731 |
| Akkermansia | Verrucomicrobiaceae | Verrucomicrobia | 0.07 ± 0.05 | 1.04 ± 1.03 | 0.26 ± 0.17 | 0.02 ± 0.01 | 0.9137 | 0.8425 | 0.2749 |

[1]Data presented as mean ± SEM. Only genera with mean relative abundances ≥ 0.05% were analyzed. *P*-values were obtained using PROC MIXED procedure of SAS with HMO, BMOS and interaction of HMO and BMOS (HMO×BMOS) as fixed effects and replicate as a random effect. Tukey post hoc test was applied when the interaction is significant. [a,b] When interaction is significant, labeled means in a row without a common letter differ, $P < 0.05$. AC, ascending colon; Uncla, Unclassified. Statistically significant _*P* values are shown in bold.[2]Taxa that have brackets around the names are proposed taxonomies that Greengenes recommend based on the whole genome phylogeny, but not officially recognized by Bergey's manual of systematic bacteriology.

**Table S3.** Relative abundances of bacterial genera detected in feces of piglets fed different diets[1,2].

| Genus | Family | Phylum | CON ($n$ = 10) | HMO ($n$ = 12) | BMOS ($n$ = 12) | HMO+BMOS ($n$ = 10) | *P* value | | |
|---|---|---|---|---|---|---|---|---|---|
| | | | | | | | HMO | BMOS | HMO×BMOS |
| Bifidobacterium | Bifidobacteriaceae | Actinobacteria | 0.03 ± 0.02 | 0.03 ± 0.01 | 0.07 ± 0.04 | 0.29 ± 0.28 | 0.3527 | 0.1887 | 0.9890 |
| Collinsella | Coriobacteriaceae | Actinobacteria | 0.06 ± 0.02 | 0.10 ± 0.02 | 0.06 ± 0.02 | 0.06 ± 0.02 | 0.2793 | 0.2591 | 0.3938 |
| Uncla Coriobacteriaceae | Coriobacteriaceae | Actinobacteria | 0.72 ± 0.28 | 2.32 ± 0.91 | 0.89 ± 0.48 | 0.65 ± 0.34 | 0.2332 | 0.1302 | 0.0869 |
| Butyricimonas | [Odoribacteraceae] | Bacteroidetes | 2.93 ± 0.92 | 4.77 ± 0.81 | 3.80 ± 0.69 | 3.18 ± 0.78 | 0.3099 | 0.7064 | 0.0993 |
| [Prevotella] | [Paraprevotellaceae] | Bacteroidetes | 0.14 ± 0.07 | 0.03 ± 0.01 | 0.23 ± 0.17 | 0.19 ± 0.09 | 0.5440 | 0.3402 | 0.3118 |
| Uncla [Paraprevotellaceae] | [Paraprevotellaceae] | Bacteroidetes | 0.80 ± 0.44 | 0.21 ± 0.10 | 0.58 ± 0.21 | 1.19 ± 0.54 | 0.7582 | 0.1570 | 0.0943 |
| Bacteroides | Bacteroidaceae | Bacteroidetes | 31.3 ± 6.15[ab] | 17.16 ± 4.57[c] | 19.26 ± 4.07[bc] | 33.44 ± 5.71[a] | 0.9027 | 0.5736 | **0.0038** |
| Parabacteroides | Porphyromonadaceae | Bacteroidetes | 5.61 ± 2.59 | 2.50 ± 0.89 | 2.68 ± 0.64 | 4.85 ± 2.48 | 0.6477 | 0.9216 | 0.0884 |
| Prevotella | Prevotellaceae | Bacteroidetes | 1.46 ± 1.22 | 0.69 ± 0.29 | 1.13 ± 0.40 | 1.03 ± 0.78 | 0.5111 | 0.7026 | 0.7647 |
| Uncla Prevotellaceae | Prevotellaceae | Bacteroidetes | 0.09 ± 0.04[b] | 0.23 ± 0.08[ab] | 0.27 ± 0.09[a] | 0.11 ± 0.05[ab] | 0.9519 | 0.4988 | **0.0125** |
| Alistipes | Rikenellaceae | Bacteroidetes | 0.56 ± 0.17 | 0.56 ± 0.18 | 0.72 ± 0.30 | 0.66 ± 0.28 | 0.7920 | 0.7286 | 0.9409 |
| Uncla Rikenellaceae | Rikenellaceae | Bacteroidetes | 2.13 ± 0.60 | 2.92 ± 0.97 | 2.89 ± 1.08 | 1.73 ± 0.49 | 0.8446 | 0.7288 | 0.3163 |
| Uncla S24-7 | S24-7 | Bacteroidetes | 0.98 ± 0.35 | 2.85 ± 0.84 | 4.62 ± 1.82 | 2.75 ± 1.22 | 0.3250 | 0.0520 | 0.0741 |
| Uncla Elusimicrobiaceae | Elusimicrobiaceae | Elusimicrobia | 4.02 ± 2.31 | 6.57 ± 3.19 | 4.13 ± 1.69 | 5.93 ± 2.59 | 0.5138 | 0.8268 | 0.8142 |
| Uncla [Mogibacteriaceae] | [Mogibacteriaceae] | Firmicutes | 0.29 ± 0.05 | 0.30 ± 0.07 | 0.23 ± 0.03 | 0.41 ± 0.27 | 0.9281 | 0.7002 | 0.8519 |
| Uncla Christensenellaceae | Christensenellaceae | Firmicutes | 0.21 ± 0.09 | 0.31 ± 0.11 | 0.08 ± 0.02 | 0.08 ± 0.03 | 0.6139 | **0.0014** | 0.4552 |
| Clostridium | Clostridiaceae | Firmicutes | 0.17 ± 0.08 | 0.18 ± 0.07 | 0.04 ± 0.01 | 0.24 ± 0.23 | 0.5167 | **0.0178** | 0.6133 |
| [Eubacterium] | Erysipelotrichaceae | Firmicutes | 0.30 ± 0.06 | 0.33 ± 0.08 | 0.23 ± 0.04 | 0.18 ± 0.06 | 0.8920 | 0.0748 | 0.4685 |
| Catenibacterium | Erysipelotrichaceae | Firmicutes | 0.17 ± 0.17 | 0.22 ± 0.15 | 0.03 ± 0.03 | 0 ± 0 | 0.9897 | 0.1542 | 0.4577 |
| RFN20 | Erysipelotrichaceae | Firmicutes | 0.02 ± 0.01 | 0.01 ± 0 | 0.02 ± 0.01 | 1.23 ± 1.17 | 0.2154 | 0.1610 | 0.1533 |
| Sharpea | Erysipelotrichaceae | Firmicutes | 0.38 ± 0.38 | 0 ± 0 | 0 ± 0 | 0 ± 0 | 0.2359 | 0.2359 | 0.2359 |
| Uncla Erysipelotrichaceae | Erysipelotrichaceae | Firmicutes | 0.12 ± 0.07 | 0.05 ± 0.02 | 0.03 ± 0.01 | 0.05 ± 0.03 | 0.3669 | 0.1848 | 0.4276 |
| [Ruminococcus] | Lachnospiraceae | Firmicutes | 2.81 ± 0.82 | 2.67 ± 0.78 | 4.28 ± 1.33 | 2.83 ± 0.82 | 0.2881 | 0.4047 | 0.5022 |
| Blautia | Lachnospiraceae | Firmicutes | 1.25 ± 0.72 | 1.71 ± 0.40 | 2.15 ± 0.76 | 1.58 ± 1.02 | 0.9133 | 0.9013 | 0.0948 |
| Clostridium | Lachnospiraceae | Firmicutes | 0.09 ± 0.03 | 0.07 ± 0.03 | 0.06 ± 0.01 | 0.06 ± 0.02 | 0.7759 | 0.7443 | 0.9899 |
| Coprococcus | Lachnospiraceae | Firmicutes | 0.22 ± 0.05 | 0.13 ± 0.02 | 0.19 ± 0.04 | 0.23 ± 0.07 | 0.3954 | 0.4848 | 0.1947 |
| Dorea | Lachnospiraceae | Firmicutes | 0.31 ± 0.10 | 0.31 ± 0.15 | 0.37 ± 0.16 | 0.12 ± 0.03 | 0.2895 | 0.4646 | 0.3936 |
| Uncla Lachnospiraceae | Lachnospiraceae | Firmicutes | 2.62 ± 0.69 | 3.56 ± 0.86 | 4.12 ± 0.66 | 4.59 ± 1.06 | 0.4039 | 0.0862 | 0.6425 |

**Continued**

**Table S3.** Relative abundances of bacterial genera detected in feces of piglets fed different diets (continued)[1,2].

| Genus | Family | Phylum | CON ($n$ = 10) | HMOs ($n$ = 12) | BMOS ($n$ = 12) | HMOs+BMOS ($n$ = 10) | *P* value | | |
|---|---|---|---|---|---|---|---|---|---|
| | | | | | | | HMOs | BMOS | HMOs×BMOS |
| Lactobacillus | Lactobacillaceae | Firmicutes | 6.49 ± 3.15 | 5.56 ± 3.50 | 6.45 ± 2.07 | 6.69 ± 2.25 | 0.6925 | 0.4812 | 0.8400 |
| Uncla Lactobacillaceae | Lactobacillaceae | Firmicutes | 0.08 ± 0.02 | 0.07 ± 0.02 | 0.11 ± 0.03 | 0.06 ± 0.02 | 0.0732 | 0.7757 | 0.4024 |
| Anaerotruncus | Ruminococcaceae | Firmicutes | 0.33 ± 0.07 | 0.28 ± 0.06 | 0.31 ± 0.08 | 0.51 ± 0.17 | 0.6735 | 0.5368 | 0.2393 |
| Butyricicoccus | Ruminococcaceae | Firmicutes | 0.12 ± 0.04 | 0.10 ± 0.05 | 0.06 ± 0.02 | 0.15 ± 0.11 | 0.8476 | 0.4595 | 0.4460 |
| Faecalibacterium | Ruminococcaceae | Firmicutes | 1.59 ± 0.40 | 1.79 ± 0.57 | 1.82 ± 0.83 | 0.82 ± 0.31 | 0.4552 | 0.2284 | 0.5611 |
| Oscillospira | Ruminococcaceae | Firmicutes | 4.91 ± 0.92 | 4.74 ± 1.05 | 3.10 ± 0.65 | 1.49 ± 0.24 | 0.1281 | **0.0014** | 0.3459 |
| Ruminococcus | Ruminococcaceae | Firmicutes | 1.17 ± 0.39 | 1.83 ± 0.55 | 0.67 ± 0.16 | 0.87 ± 0.31 | 0.3189 | **0.0347** | 0.9353 |
| Uncla Ruminococcaceae | Ruminococcaceae | Firmicutes | 6.46 ± 0.62 | 9.26 ± 2.15 | 3.93 ± 0.81 | 3.32 ± 0.67 | 0.7045 | **0.0006** | 0.3055 |
| Acidaminococcus | Veillonellaceae | Firmicutes | 0.15 ± 0.10 | 0.95 ± 0.35 | 2.64 ± 0.92 | 2.75 ± 1.22 | 0.3205 | **0.0014** | 0.2562 |
| Megasphaera | Veillonellaceae | Firmicutes | 0.09 ± 0.02 | 0.19 ± 0.08 | 0.49 ± 0.16 | 0.34 ± 0.11 | 0.9234 | **0.0036** | 0.2001 |
| Mitsuokella | Veillonellaceae | Firmicutes | 0.05 ± 0.05 | 1.46 ± 1.37 | 0.05 ± 0.03 | 0.59 ± 0.59 | 0.1630 | 0.6616 | 0.5541 |
| Phascolarctobacterium | Veillonellaceae | Firmicutes | 0.04 ± 0.01 | 0.05 ± 0.02 | 0.07 ± 0.02 | 0.06 ± 0.01 | 0.8277 | 0.1559 | 0.5727 |
| Uncla Veillonellaceae | Veillonellaceae | Firmicutes | 0.10 ± 0.02 | 0.38 ± 0.28 | 0.48 ± 0.13 | 0.40 ± 0.16 | 0.8091 | **0.0196** | 0.2825 |
| Uncla Fusobacteriaceae | Fusobacteriaceae | Fusobacteria | 2.35 ± 1.97 | 0.60 ± 0.38 | 1.02 ± 0.74 | 0.11 ± 0.05 | 0.1924 | 0.2579 | 0.8102 |
| Uncla Victivallaceae | Victivallaceae | Lentisphaerae | 0.08 ± 0.06 | 0.08 ± 0.03 | 0.04 ± 0.02 | 0.02 ± 0.01 | 0.8839 | 0.0676 | 0.6073 |
| Sutterella | Alcaligenaceae | Proteobacteria | 0.17 ± 0.10 | 0.14 ± 0.06 | 0.37 ± 0.24 | 0.21 ± 0.10 | 0.4979 | 0.4655 | 0.6273 |
| Campylobacter | Campylobacteraceae | Proteobacteria | 0.56 ± 0.56 | 0 ± 0 | 0.13 ± 0.13 | 0.01 ± 0.01 | 0.2266 | 0.8099 | 0.5741 |
| Bilophila | Desulfovibrionaceae | Proteobacteria | 0.13 ± 0.06 | 0.11 ± 0.02 | 0.11 ± 0.04 | 0.17 ± 0.09 | 0.6453 | 0.8760 | 0.9013 |
| Desulfovibrio | Desulfovibrionaceae | Proteobacteria | 0.54 ± 0.11 | 0.72 ± 0.15 | 0.61 ± 0.12 | 0.43 ± 0.12 | 0.8105 | 0.2440 | 0.0948 |
| Uncla Desulfovibrionaceae | Desulfovibrionaceae | Proteobacteria | 0.13 ± 0.03 | 0.09 ± 0.01 | 0.14 ± 0.03 | 0.12 ± 0.04 | 0.2737 | 0.6305 | 0.8424 |
| Escherichia | Enterobacteriaceae | Proteobacteria | 0.06 ± 0.02 | 0.40 ± 0.30 | 1.64 ± 0.68 | 0.59 ± 0.31 | 0.5550 | **0.0099** | 0.1114 |
| Uncla Enterobacteriaceae | Enterobacteriaceae | Proteobacteria | 0.01 ± 0 | 0.03 ± 0.01 | 0.13 ± 0.05 | 0.06 ± 0.03 | 0.7383 | **0.0124** | 0.0913 |
| Flexispira | Helicobacteraceae | Proteobacteria | 0.05 ± 0.04 | 0.03 ± 0.02 | 0.14 ± 0.13 | 0.01 ± 0.01 | 0.5484 | 0.7082 | 0.6329 |
| Synergistes | Synergistaceae | Synergistetes | 0.37 ± 0.16 | 1.18 ± 0.84 | 0.30 ± 0.10 | 0.66 ± 0.43 | 0.2624 | 0.4149 | 0.5058 |
| Uncla Synergistaceae | Synergistaceae | Synergistetes | 0.06 ± 0.02 | 0.10 ± 0.03 | 0.04 ± 0.01 | 0.07 ± 0.04 | 0.2854 | 0.1615 | 0.6841 |
| Akkermansia | Verrucomicrobiaceae | Verrucomicrobia | 1.05 ± 0.50 | 1.49 ± 1.35 | 2.01 ± 1.16 | 0.48 ± 0.36 | 0.2868 | 0.9263 | 0.5572 |

[1]Data presented as mean ± SEM. Only genera with mean relative abundances ≥ 0.05% were analyzed. *P*-values were obtained using PROC MIXED procedure of SAS with HMO, BMOS and interaction of HMO and BMOS (HMO×BMOS) as fixed effects and replicate as a random effect. Tukey post hoc test was applied when the interaction is significant. [a,b,c] When interaction is significant, labeled means in a row without a common letter differ, $P < 0.05$. AC, ascending colon. Uncla, Unclassified. Statistically significant _*P* values are shown in bold. [2]Taxa that have brackets around the names are proposed taxonomies that Greengenes recommend based on the whole genome phylogeny, but not officially recognized by Bergey's manual of systematic bacteriology.

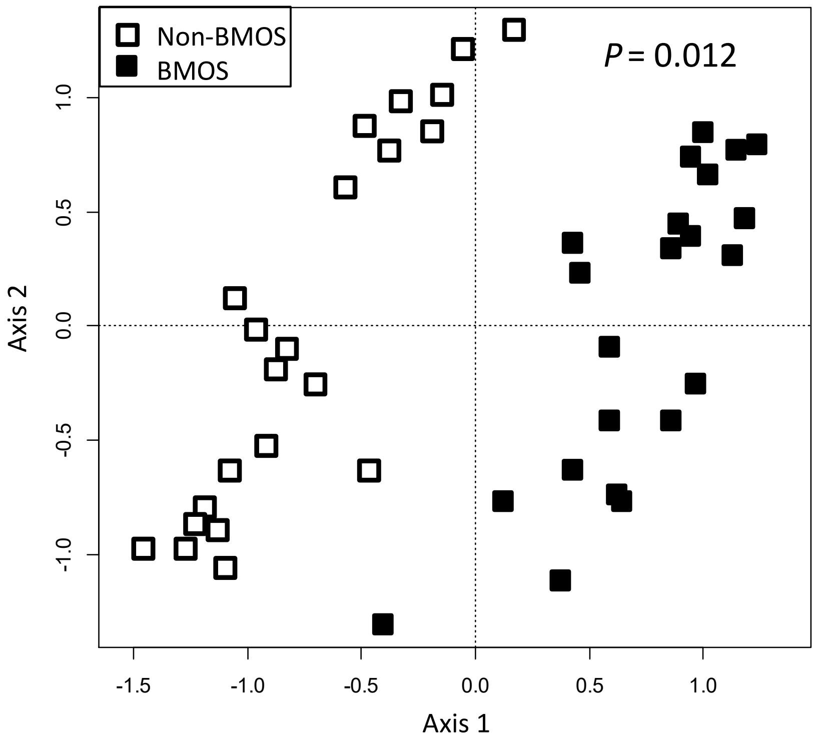


**Figure S1.** Distance-based redundancy analysis based on unweighted UniFrac distances generated from ascending colon contents of piglets fed diets with BMOS (BMOS and HMO+BMOS groups) and without BMOS (CON and HMO groups). The statistical model contained only BMOS. Supplementation of BMOS modulated the overall microbiota of AC contents regardless the presence of HMO ($P = 0.012$).
